# A single point mutation on FLT3L-Fc protein increases the risk of immunogenicity

**DOI:** 10.3389/fimmu.2025.1519452

**Published:** 2025-02-13

**Authors:** Dan Qin, Qui Phung, Patrick Wu, Zhaojun Yin, Sien Tam, Peter Tran, Adel M. ElSohly, Joshua Gober, Zicheng Hu, Zhenru Zhou, Sivan Cohen, Dongping He, Travis W. Bainbridge, Christopher C. Kemball, Jonathan Zarzar, Alavattam Sreedhara, Nicole Stephens, Jérémie Decalf, Christine Moussion, Zhengmao Ye, Mercedesz Balazs, Yinyin Li

**Affiliations:** ^1^ Biochemical and Cellular Pharmacology, Genentech Inc., South San Francisco, CA, United States; ^2^ Proteomic and Genomic Technologies, Genentech Inc., South San Francisco, CA, United States; ^3^ BioAnalytical Sciences, Genentech Inc., South San Francisco, CA, United States; ^4^ Protein Chemistry, Genentech Inc., South San Francisco, CA, United States; ^5^ Pharmaceutical Development, Genentech Inc., South San Francisco, CA, United States; ^6^ Analytical Development & Quality Control, Genentech Inc., South San Francisco, CA, United States; ^7^ Cancer Immunology, Genentech Inc., South San Francisco, CA, United States

**Keywords:** anti-drug antibodies, immunogenicity, FLT3L-Fc, in silico prediction, *in vitro* cellular assay, T cell proliferation, DC internalization, MAPPS

## Abstract

**Introduction:**

As a crucial asset for human health and modern medicine, an increasing number of biotherapeutics are entering the clinic. However, due to their complexity, these drugs have a higher potential to be immunogenic, leading to the generation of anti-drug antibodies (ADAs). Clinically significant ADAs have an impact on pharmacokinetics (PK), pharmacodynamics (PD), effectiveness, and/or safety. Thus, it is crucial to understand, manage and minimize the immunogenicity potential during drug development, ideally starting from the molecule design stage.

**Methods:**

In this study, we utilized various immunogenicity risk assessment methods, including *in silico* prediction, dendritic cell internalization, MHC-associated peptide proteomics, *in vitro* HLA peptide binding, and *in vitro* T cell proliferation, to assess the immunogenicity risk of FLT3L-Fc variants.

**Results:**

We identified a single point mutation in the human FLT3L-Fc protein that introduced highly immunogenic T cell epitopes, leading to the induction of T cell responses and thereby increasing the immunogenicity risk in clinical settings. Consequently, the variant with this point mutation was removed from further consideration as a clinical candidate.

**Discussion:**

This finding underscores the necessity for careful evaluation of mutations during the engineering of protein therapeutics. The integration of multiple immunogenicity risk assessment tools offers critical insights for informed decision-making in candidate sequence design and therapeutic lead selection.

## 1 Introduction

Over the past few decades, biotherapeutics, particularly monoclonal antibodies, have significantly advanced our ability to treat complex diseases due to their high specificity and well-characterized mechanisms of action (1, 2). However, a major challenge associated with these highly engineered biologics is their potential to induce immunogenicity (3–5). The clinically significant immunogenicity can profoundly impact both the efficacy and safety of biotherapeutics (6–9). When the immune system identifies a therapeutic protein as foreign, it can produce anti-drug antibodies (ADAs). These ADAs can neutralize the therapeutic agent and alter its pharmacokinetics and biological effects, thereby diminishing drug efficacy. In severe instances, the immune response may provoke hypersensitivity reactions, which can be life threatening (10–12). A review of the FDA’s clinical pharmacology data for biologics approved before February 2015 revealed that 89% of cases reported the incidence of ADAs, 60% reported neutralizing activity, and 26% indicated that immunogenicity affected pharmacokinetics. Approximately 50% of these products experienced an impact on efficacy, and 60% reported an effect of immunogenicity on safety (13). Consequently, the development of biologics with reduced immunogenicity risks is of paramount importance. In accordance with FDA and EMA (European Medicines Agency) guidelines, immunogenicity monitoring is a regulatory requirement in clinical studies (14, 15). Immunogenicity assessment and risk mitigation are integral components of biotherapeutic development, spanning from lead identification and optimization to early development and clinical trials, thereby ensuring patient safety during post-marketing surveillance.

The generation of ADAs involves multiple steps of cellular responses of the innate and adaptive immune systems (16). Following the administration of a biotherapeutic, antigen-presenting cells (APCs), such as dendritic cells (DCs), internalize and process the therapeutic protein into antigen-specific peptides. These peptides are then presented on major histocompatibility complex (MHC) class II molecules (pMHCII) to T cell receptors (TCRs) on CD4^+^ T cells, promoting cytokine release and eliciting T cell activation and proliferation. Activated T cells subsequently stimulate B cells, ultimately leading to the production of ADAs. Recently, a suite of assays has been developed to map these distinct steps, ensuring a comprehensive immunogenicity risk assessment during drug development (17). *In silico* prediction algorithms are employed to forecast potential T cell epitopes (18, 19). DC internalization assays evaluate the uptake of the drug by APCs (20, 21). The MHC-associated peptide proteomics (MAPPs) assay identifies antigen-specific peptides that bind to MHCII molecules (22, 23). The *in vitro* recombinant HLA peptide binding assay assesses the binding efficiency between MHCII proteins and the peptides of interest. Furthermore, T cell responses can be evaluated using *in vitro* T cell activation and proliferation assays (24–26), while the DC:T cell co-culture assay is utilized for immunomodulators (27). A recent study has demonstrated the utility of these assays in non-clinical immunogenicity risk assessment for bispecific IgG1 antibodies, thereby providing valuable insights for antibody design and optimization during drug development (28).

**Figure 1:**
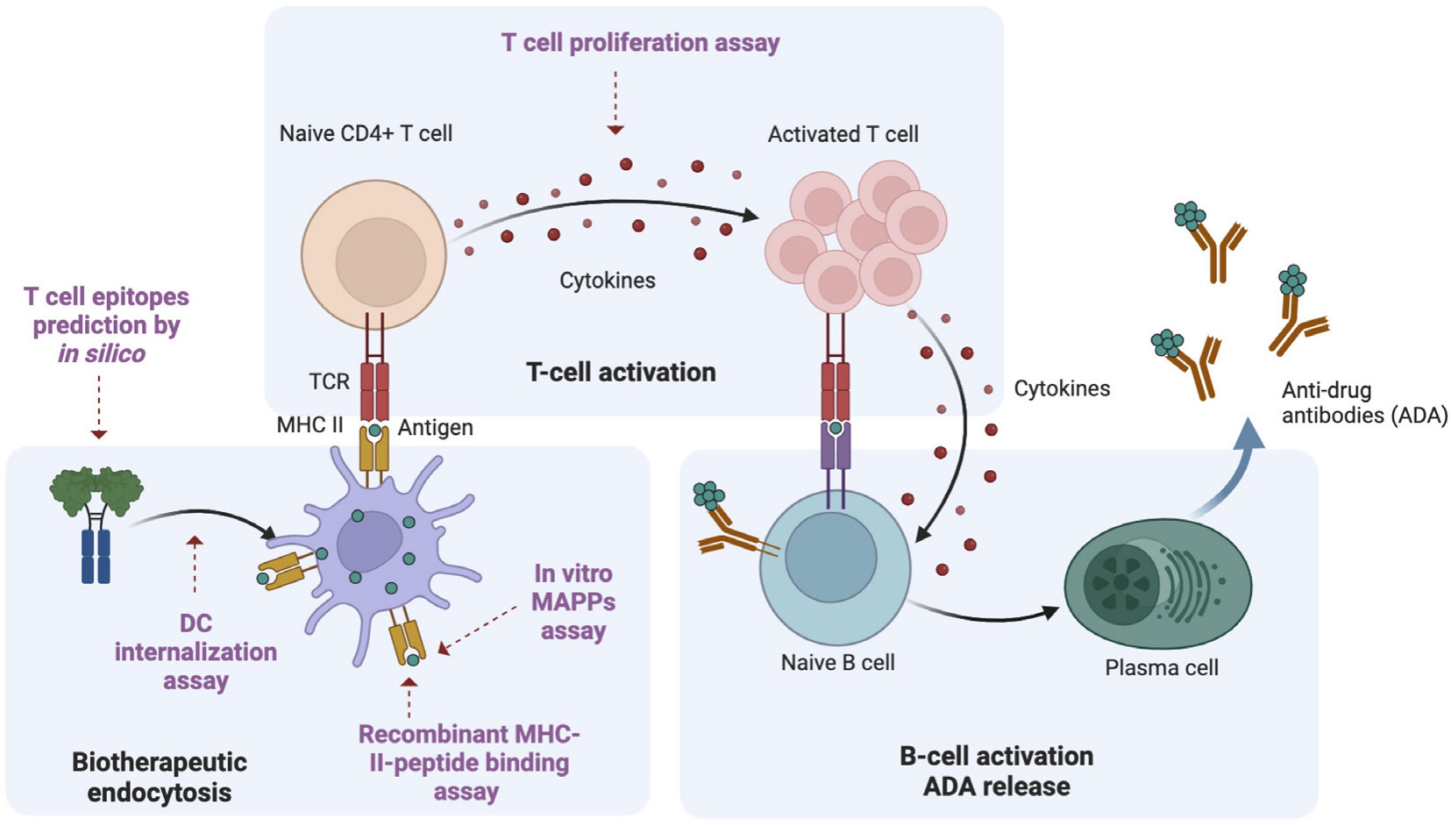
Overview of ADA generation pathway. Using a Fc fusion protein as an example protein therapeutic, there are three key steps involved in the ADA response. Biotherapeutic endocytosis by antigen presenting cells (APCs): APCs, such as dendritic cells (DCs) uptake the biotherapeutic and process it into antigen-specific peptides; Activation of naive CD4^+^ T cells: these antigen-specific peptides are presented to T cell receptors on MHCII, leading to the activation of naive CD4^+^ T cells; B cell activation and ADA production: activated CD4^+^ T cells will further induce B cell activation, maturation and differentiation. Plasmablasts eventually produce ADA. Multiple methods for immunogenicity risk assessment map different steps in the ADA generation pathway. In this study, we utilized different methods to characterize the immunogenicity risk: *in silico* prediction to predict the peptides presented by MHCII; DC internalization to assess drug uptake by APCs; MAPPs to identify antigen-specific peptides binding to MHCII, *in vitro* recombinant MHCII peptide binding assay to evaluate the efficiency of binding between MHCII proteins and the peptides of interest; T cell proliferation assay to evaluate T cell response. This figure was adapted from “Anti-drug antibodies (ADA) Immunogenicity Assessment” by BioRender.com (2024). Retrieved from https://app.biorender.com/biorender-templates.

It is important to recognize that the immunogenicity of a given therapeutic is multifactorial (29), but T cell epitopes play a major role in eliciting immunogenic responses (17, 30, 31). The development of biotherapeutics with optimal efficacy and minimal toxicity necessitates the incorporation of various mutations into the primary sequence during antibody design. However, these modifications may inadvertently introduce novel T cell epitopes (28, 32), and an increase in the number of presented T cell epitopes generally correlates with a heightened risk of immunogenicity (22, 23, 33). In this study, we used in silico epitope prediction in conjunction with a suite of *in vitro* assays to evaluate the immunogenicity risk of FLT3L-Fc variants during the lead selection process (**Figure 1**). This serves as a case study to highlight the importance of T cell epitopes in immunogenicity. For in silico epitope prediction, we employed two peptide presentation prediction models, NetMHCIIpan-3.2 (34) and NetMHCIIpan-4.0 (19). NetMHCIIpan-4.0, which incorporates extensive ligand data from mass spectrometry, outperforms NetMHCIIpan-3.2, enabling more accurate identification of potential T cell epitopes (35). Recombinant FLT3L, such as CDX-301, and FLT3 agonistic Fc fusion proteins have demonstrated the ability to expand dendritic cells (DCs) and hematopoietic precursors in healthy human volunteers (36, 37), which suggest that the FLT3L pathway has the potential to promote T cell-mediated anti-tumor activity. The two wild-type FLT3L proteins, containing native sequence, have been well tolerated, with low immunogenicity responses reported in phase 1 and 2 trials (38, 39). FLT3L-Fc (Genentech, Inc., South San Francisco, CA, USA) is a half-life extended fusion protein comprising the human native FLT3 ligand extracellular domain and an IgG1 Fc, which has been modified to attenuate effector function. The therapeutic strategy involves utilizing FLT3L-Fc to expand conventional DC1s (cDC1s) to enhance antitumor immunity (40). During molecule assessment, we identified a tryptophan residue in the FLT3 ligand sequence that is susceptible to oxidative degradation to varying extents under biologically relevant conditions (41, 42). To address the oxidation liability of the wild-type (WT) FLT3L-Fc molecule, we engineered a mutant FLT3L-Fc variant by substituting Trp144 with aspartic acid in the native ligand domain. Intriguingly, our data indicate that this single point mutation introduces a potential new T cell epitope that binds to the promiscuous human MHCII, significantly eliciting T cell responses and thereby increasing the immunogenicity risk. This case study underscores the practical application of multiple immunogenicity assessment tools, which can be synergistically employed to generate a comprehensive report, thereby informing decision-making in therapeutic lead selection.

## 2 Materials and methods

### 2.1 Oxidative stress assay

1 mg of FLT3L-WT protein was oxidatively stressed by incubating with 1 mM AAPH (2,2′-Azobis (2-amidinopropane) dihydrochloride) (Cayman Company, Cat# 0554100-6) in low-ionic strength histidine-acetate buffer, pH 5.5, for 16 h at 40°C. After 16 h incubation, the reaction was quenched with excess methionine (Met) at a ratio 20:1 (Met to AAPH). Control samples were prepared by the addition of water in place of AAPH. Control and stress samples were buffer-exchanged prior to analysis by LC-MS/MS peptide mapping.

### 2.2 LC-MS/MS peptide mapping

A 250 µg sample of control and stressed protein was reduced with 20 mM DTT in 6M guanidine hydrochloride, 360 mM Tris, and 2 mM EDTA at pH 7.0 for 1 h. After the reduction, samples were cooled to room temperature and iodoacetic acid was used for alkylation with a final concentration of 50 mM for 15 min in darkness. Samples were then buffer exchanged into the digestion buffer (composed of 50 mM Tris, 2 mM CaCl_2_, pH 7.5) and subjected to trypsin digestion for 1 h at 37°C (where the enzyme to substrate ratio was 1:20 by weight). The reaction was terminated by adding a final concentration of 2.2 mM L-Methionine along with 3% formic acid. Samples were then analyzed using a Vanquish UHPLC (Thermo Fisher) paired with an Exploris240 (Thermo Fisher) mass spectrophotometer. Separation was performed on an Acquity UPLC peptide CSH C18 column consisting of 1.7 µm, 130 Å particles (Waters), at a flow rate of 0.2 mL/min at 77°C. The mobile phase A was 0.1% formic acid in water and mobile phase B was 0.1% formic acid in acetonitrile. The separation gradient was set as follows: 1% mobile phase B for 2 minutes, 1-13% mobile phase B over 5 minutes, 13-35% mobile phase B over 35 minutes, 35-95% mobile phase B over 2 minutes, and then 95% mobile phase B for 2 minutes. Mass spectrometry data was collected in positive ion mode using a Top 8 data dependent scan with a resolution of 60000 for MS scans and 30000 for MS2 scans. An external calibration of the instrument was performed prior to sample analysis. Mass spectrometry data was processed using PMI-Byos software (Protein Metrics) using the PTM workflow. Relative quantitation was calculated using the top two charge states for both the native tryptic peptide and the modified counterpart. The percent of tryptophan oxidation following oxidative stress were then calculated.

### 2.3 OCI-AML5 proliferation assay

White 96 well plates (Corning) were coated overnight at room temperature (RT) with 75 μL poly-L-ornithine solution (Millipore), washed 3 times with PBS and dried. OCI-AML5 cells were seeded at 3000 cells/well in 75 μL assay media (RPMI 1640, 5% heat inactivated FBS, 1 × Glutamax). FLT3L-Fc was serially diluted 1:10 with 10 nM starting concentration for 9 points. 75 μL of FLT3L-Fc diluted solutions were added to assay wells in triplicate. Maximum and neutral control wells included 10 nM FLT3L-Fc-WT or assay media alone. Assay plates were incubated at 37°C, 5% CO_2_ for 7 days. 50 μL of CellTiter-Glo Luminescent cell viability reagent (Promega) was added. Plates were incubated for 10 min and luminescence was measured with a Tecan Infinite M1000 Microplate Reader. Raw values were normalized to the maximum and neutral control wells. Dose response curves were plotted, and 50% effective concentration (EC50) values were determined with GraphPad Prism using a four-parameter logistic fit.

### 2.4 *In silico* epitope prediction

Potential immunogenic T cell epitopes were computationally predicted using NetMHCIIpan-3.2 (34) and NetMHCIIpan-4.0 (35) for the nine common HLA-DR alleles: HLA-DRB1*01:01, HLA-DRB1*03:01, HLA-DRB1*04:01, HLA-DRB1*07:01, HLA-DRB1*08:01, HLA-DRB1*09:01, HLA-DRB1*11:01, HLA-DRB1*13:01, and HLA-DRB1*15:01. For each FLT3L-Fc variant, the FLT3L region was computationally digested into all possible overlapping 12-20-mer peptides. These peptides were considered to be strong binders or presented on HLA-DR if the percentile rank score for the predicted binding core (a 9-mer peptide) was within the top 10% (43, 44). Among these binding cores, likely tolerized epitopes were excluded if their sequences were identically aligned to 9-mer peptides from the human reference proteome using PEPMatch (45). Among the remaining binding cores, those that were predicted to bind to >1 HLA-DR molecules were considered potentially immunogenic CD4 T cell epitopes (43, 44). For each epitope, global population coverage was calculated using allele frequencies from the Immune Epitope-Database (IEDB) population coverage tool (46).

### 2.5 DC internalization assay

DC generation and loading was conducted following a previously published method (47). In brief, monocytes were isolated from human blood from anonymous healthy volunteers participating in the Genentech blood donor program, after receiving a written, informed consent from the Western Institutional Review Board. Monocytes were differentiated into immature DCs using interleukin-4 (IL-4) at 50 ng/ml and granulocyte-macrophage colony-stimulating factor (GM-CSF) at 3 ng/ml for five days. After incubation with 100 µg/mL of the test antibody and 1 µg/mL of lipopolysaccharide (Sigma) for 24 h, the DCs were harvested and washed with fluorescence-activated cell sorter (FACS) buffer (phosphate-buffered saline containing 2% fetal bovine serum and 0.02% sodium azide). For external staining, cells were stained with goat F(ab’)_2_ anti-human IgG (H+L) conjugated to AlexaFluor-647 (Jackson ImmunoResearch Laboratories) for 30 min on ice, washed three times with FACS buffer and then fixed with 4% paraformaldehyde for 10 min. The cells were washed and resuspended in the FACS buffer before analysis. For total staining, cells were permeabilized and fixed with Fixation and Permeabilization Solution (BD Biosciences), washed three times with Permeabilization Solution prior to staining with the same goat F(ab’)_2_ anti-human IgG (H+L) conjugated to AlexaFluor-647 for 30 min on ice. Cells were washed with permeabilization wash buffer and resuspended in FACS buffer before analysis. The cells were analyzed with an Attune NxT flow cytometer (Invitrogen). The data was analyzed using FlowJo (BD Biosciences, version 10.7.2) and plotted in Prism (v9.5.1). The internalization index was calculated by subtracting the fluorescence signal of the median fluorescence intensity (MFI) of externally stained cells from the MFI of the total stained cells.

### 2.6 FLT3 receptor staining in DC

The mature monocyte derived DC (MoDC) was obtained by incubating immature MoDC with 1 µg/mL of LPS overnight. To stain the surface FLT3 receptors on both immature and mature DCs, 20 μL of PE conjugated anti-human CD135 antibody (BD PharmingenTM) or isotype control (BD PharmingenTM) were added to 200 μL of cells (10^6^ cells/mL) respectively and incubated on ice for 30 minutes. The cells were washed and resuspended in the FACS buffer (phosphate-buffered saline containing 2% fetal bovine serum and 0.02% sodium azide) before analysis. The cells were analyzed with an Attune NxT flow cytometer (Invitrogen). The data was analyzed using FlowJo (BD Biosciences, version 10.7.2) and plotted in Prism (v9.5.1).

### 2.7 *In vitro* T cell proliferation assay

Cryopreserved peripheral blood mononuclear cells (PBMCs) from 48 healthy donors were thawed at 37°C water bath using Aim-V media (Gibco, Cat# 12055091) supplemented with 3% human serum (Sigma-Aldrich, Cat# H3667). Cells were then seeded in a 24-well plate with Lonza X-vivo15 media (Lonza, Cat# 04-418Q) at a concentration of 4X10^6^ cells/mL, with each well containing 1 mL medium. Following seeding, testing agents were then added to the designated wells at a final concentration of 50 ug/mL for each group. All cells were cultured in a 37°C incubator with 5% CO_2_ for five days. On day six, BrdU reagent (BD Bioscience, Cat# 557891) was added to the cells, and they were further cultured for an additional 24 hours. The following day, cells were harvested and then stained with Aqua Live/Dead (Thermo Fisher, Cat# L34957), anti-BV421-CD3 (BD Bioscience, Cat# 562426), anti-PE-Cy-7-CD4 (BD Bioscience, Cat# 557852), anti-APC-CD14 (BD Bioscience, Cat# 555399), anti-APC-CD19 (BD Bioscience, Cat# 555415) antibodies. After surface staining, cells were washed with 0.5% BSA-PBS and then fixed and permeabilized by Cytofix/Cytoperm buffer (BD Bioscience, Cat# 554714) according to the manufacturing protocol. Cells were then stained with anti-BrdU (BD Bioscience, Cat# 557891) antibody followed by flow cytometry analysis on Attune (Invitrogen™). FACS files were processed using FlowJo software (Tree Star, Inc.; Ashland, OR) and downstream data analysis was performed by R studio.

### 2.8 MAPPs assay

#### 2.8.1 MAPPs sample generation

CD14^+^ monocytes were isolated from 10 healthy human donor PBMCs collected from whole blood and leukapheresis. Monocytes were cultured in RPMI media supplemented with 10% FBS, L-glutamine, non-essential amino acids, sodium pyruvate, and gentamycin containing GM-CSF and IL-4. After five days in culture, the monocytes were differentiated into immature DCs (iDCs). The iDCs were treated with 100 ug/mL protein therapeutic and matured with 1ug/mL lipopolysaccharide (LPS) for 16 hrs. On day 6, the mature DCs (mDCs) were harvested, counted, and washed in Dulbecco’s PBS. Cells were flash frozen and stored at -80°C until ready for downstream processing.

#### 2.8.2 MAPPs sample processing

Frozen DC pellets (1-1.5×10^6^ cells/pellet) treated with protein therapeutics were lysed in 1 mL Pierce Lysis buffer (Thermo #8778), protease inhibitor (Roche #11836170001), 25 ug/mL DNase (Roche #10104159001) for 30 min at 4°C. Lysates were cleared by centrifugation at 20,000xg for 30 min at 4°C then transferred to a deep well plate. HLA-DR was then immunoprecipitated from the lysates using in-house purified L243-antibody (ATCC HB-55) covalently cross-linked to Protein A cartridges (Agilent #G5496-6000). Briefly, using the Agilent Assay map Bravo platform, lysates were loaded onto the L243-bound Protein A cartridges, washed with 20 mM Tris-HCl buffers containing varying salt concentrations, and eluted with 0.1 M acetic acid/0.1% trifluoroacetic acid.

IP eluent samples were desalted, and peptides were injected onto a trapping column and eluted over a 75 um analytical column packed with Luna C18 resin (Phenomenex). Chromatographic separation over a 1hr gradient was performed at a flow rate 350 nL/min using a Waters NanoAcquity LC interfaced to a ThermoFisher Fusion Lumos mass spectrometer at a spray voltage of 1.95 kV. Mass spectral data were acquired using a method comprising of one full MS scan (300-1500 m/z) in the Orbitrap at resolution of 60,000 M/ΔM at m/z 400 followed by higher-energy C-trap dissociation (HCD) of the top 10 most abundant ions detected in the full MS scan at FWHM resolution of 15,000, in a cycle repeated throughout the LC gradient. The top 10 peptides were selected for MS/MS with a dynamic exclusion of 15 s.

Tandem mass spectral results were submitted for database searching using PEAKS Online v11 (48) (BSI) against a database containing sequences from the UniProtKB/Swiss-Prot Homo sapiens proteome and protein therapeutics (concatenated forward and reversed plus common laboratory contaminants). The data was searched with no enzyme specificity, variable modification of oxidation (Met, +15.995 Da), deamidation (Asn and/or Gln, +0.984), carbamidomethylation (Cys,+57.042), 3 maximum variable post-translational modifications per peptide, 10 ppm precursor mass tolerance, and fragment ion tolerance at 0.02 Da. Peptide spectral matches were filtered using the decoy fusion method to an estimated false discovery rate (FDR) of 1%.

MAPPs results were represented in a heatmap spanning across the sequence of the protein using the R visualization package, PepMapViz (49). Briefly, peptides corresponding to the biotherapeutic(s) identified from the database search were further filtered by length, 11 to 30 amino acids. The area under the curve of every peptide sequence with signal above the noise is calculated from the corresponding mass spectrum. The maximum area of each unique peptide sequence is selected. The heatmap is colored by the area of epitopes, which is determined by the summation of area of each unique sequence in a peptide cluster; where a cluster is defined as the presented T cell epitopes of varying lengths which share the same MHCII binding core.

### 2.9 Binding of peptides to recombinant MHCII assay

#### 2.9.1 Protein expression and purification

MHCII proteins containing C-terminal Fos/Jun leucine zippers were expressed in Chinese Hamster Ovary (CHO) cells. Recombinant MHCII was purified from cell culture supernatant by nickel-NTA chromatography using a HisTrap Excel column (Cytiva). Fractions containing pMHCII were pooled and concentrated using a 10K MWCO Amicon centrifugal filter (EMD Millipore). The concentrated protein solution was then purified by size exclusion chromatography using a Superdex 200 10/300 GL column (Cytiva). Protein purity was determined to be >90% by SDS-PAGE.

#### 2.9.2 Peptides for 2D-LCMS screening

The peptides used for 2D-LCMS evaluation were purchased from Elim Biopharma at >95% purity. 4 mM stock solutions of each peptide were prepared in ethylene glycol and stored at -20°C.

#### 2.9.3 TEV cleavage

1 mL of 2-5 mg/mL pMHCII in PBS pH 7.4 was incubated at room temperature with 30 μg/mL of the TEV S219V variant (50). The reaction progress was monitored by LCMS until cleavage was complete.

#### 2.9.4 Peptide exchange

A master mix of 17.8 μM protease-cleaved MHCII was prepared in a buffer containing 44.5 mM sodium acetate pH 5.0, 167 mM NaCl, 4.45 mM EDTA, and 2.23 mM NaN_3_. A 25-fold molar excess of peptide from a 4 mM stock solution in ethylene glycol was added to the MHCII master mix to give a final composition of 16 μM MHCII, 400 μM peptide, 40 mM sodium acetate pH 5, 150 mM NaCl, 4 mM EDTA, 2 mM NaN_3_, and 0.50 mM TCEP. Peptide exchange was performed by incubating the mixture at 37°C for 60-72 h. After incubation, an equal volume of 50 mM Tris pH 8.0, 150 mM NaCl was added to neutralize the mixture prior to 2D-LCMS analysis.

#### 2.9.5 2D-LCMS

2D-LCMS is used to evaluate the efficiency of *in vitro* peptide exchange with MHCII proteins and peptides of interest. In the first dimension, pMHCII was separated on an analytical size exclusion column (Zenix SEC-100, 100 Å, 3 μm, 4.6 x 150 mm) from excess peptide in the reaction mixture and then collected in a sampling loop. In the second dimension, the purified pMHCII collected in the sampling loop was injected onto a reversed-phase column (Agilent PLRP-S, 1000 Å, 8 μm, 50 x 2.1 mm) to separate bound peptides from the MHCII complex. 20 μL of approximately 0.5 mg/mL neutralized pMHCII mixture were used for analysis. In the first dimension, the protein was eluted at 0.4 mL/min in 25 mM Tris pH 8.0, 150 mM NaCl, 2 mM NaN_3_ for 11 min. In the second dimension, peptide(s) and MHCII were separated using a gradient of 5–50% mobile phase B in 4.7 min at 0.55 mL/min with the column heated to 80°C (mobile phase A = 0.05% TFA in water, mobile phase B = 0.05% TFA in acetonitrile). Eluted peptides and proteins from the second dimension were then sent to a mass spectrometer for electrospray ionization and mass detection in positive ion mode (Agilent 6224 ESI-TOF LCMS). LCMS data analysis was performed using Agilent MassHunter Quantitative Analysis software. Combined extracted ion chromatograms (EICs) were prepared using the extracted intensities for the [M+H]+, [M+2H]2+, and [M+3H]3+ ions. Query peptides with a combined EIC intensity of >5 ×10^3^ were considered to be MHCII binders.

### 2.10 Bacterial sequence similarity search

To determine whether any of the *in silico* predicted epitopes resembled microbial sequences, we used NCBI protein BLAST (BLASTP) to search the non-redundant protein sequences (nr) database (51). We analyzed predicted epitopes from FLT3L-Fc-WT and FLT3L-Fc-W144D (e.g. LVALKPWIT and LVALKPDIT, respectively) using BLASTP with the following the short input sequence settings: word size = 2, expect value = 200000, hitlist size = 100, gap open costs = 9, gap extension cost = 1, matrix = PAM30, window Size = 40 and threshold = 11. The search results were filtered to identify 8- and 9-mer sequences that were identical to bacterial protein sequences.

## 3 Results

### 3.1 Selection of FLT3L-Fc mutein to mitigate oxidation liability

**Figure 2:**
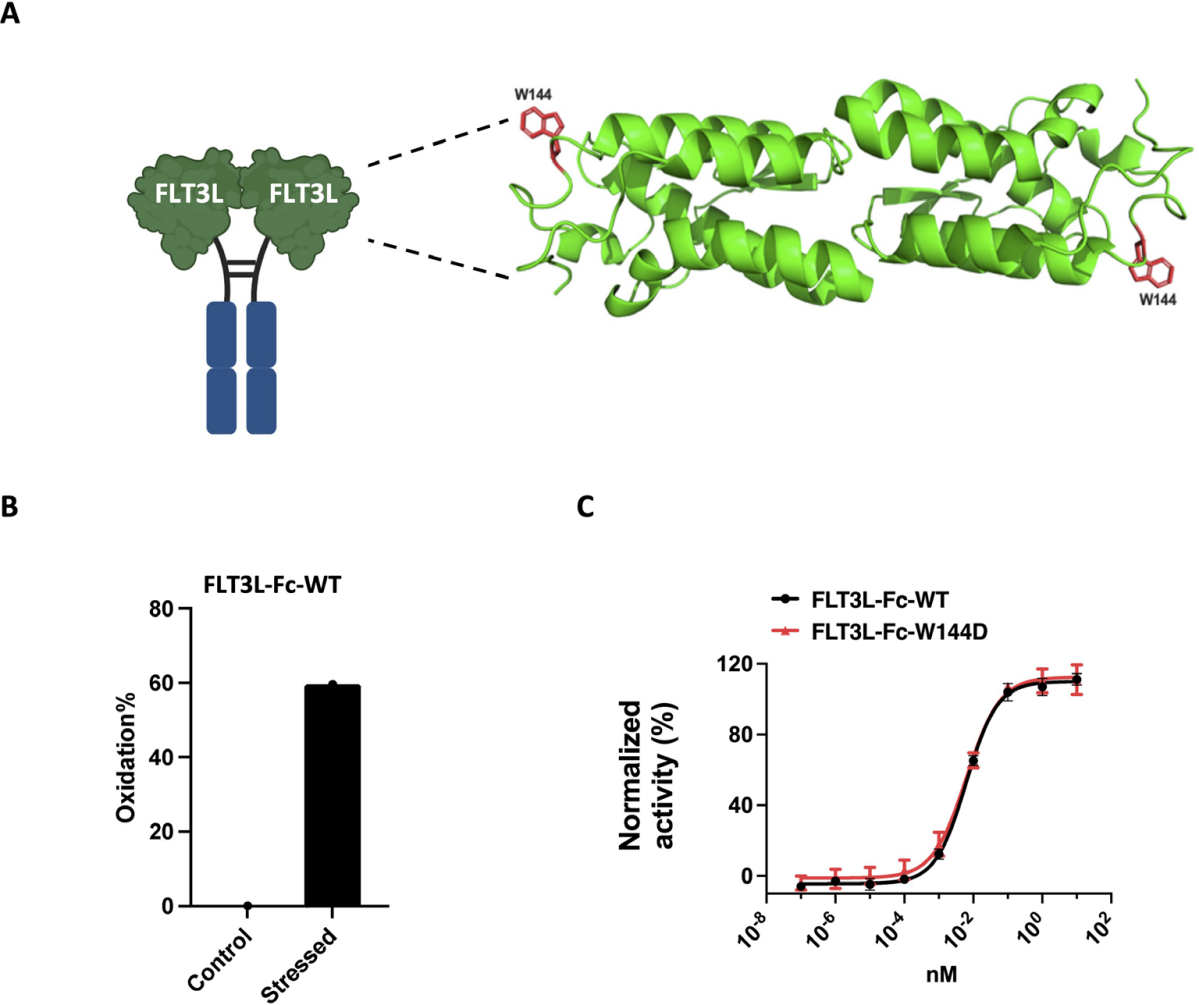
Selection of FLT3L-Fc mutein to mitigate oxidation liability. **(A)** Illustration of the location of W144 on FLT3L-Fc. Structure of the four-helix bundle extracellular domain of the FLT3L homodimer (PDB code 3QS7). The residue W144 is shown in red. **(B)** Oxidation percentages of control and AAPH stressed FLT3L-Fc were assessed by LC-MS/MS peptide mapping. **(C)** Proliferation of OCI-AML5 cells *in vitro*. A representative dose response curve (average ± standard deviation (SD) of six replicate values) from two assays is shown. Data were normalized to 10 μg/mL FLT3L-Fc-WT as the maximum (100%) response and the assay media alone control as the minimum (0%) response.

Given the limitations associated with the rapid clearance of FLT3 ligand in clinical settings, we engineered a FLT3L-Fc fusion protein to extend its half-life and enhance its pharmacokinetic profile (40). During molecule assessment of FLT3L-Fc, we identified a tryptophan residue (W144) within the FLT3L portion of the protein (**Figure 2A**) that exhibited high susceptibility to oxidation when stressed with AAPH (2,2′-Azobis (2-amidinopropane) dihydrochloride) (**Figure 2B**). To address this oxidation liability, which may impact the biological activities of the molecule, we computationally mutated W144 to other residues except Methionine and Cysteine without oxidation risk. To reduce the risk of generating new T cell epitopes, we aimed to identify the muteins with the lowest immunogenicity risk. Using NetMHCIIpan-3.2 (34), the FLT3L-Fc variant with the lowest predicted immunogenicity risk had W144 replaced by aspartic acid (W144D mutein, **Supplementary Table S1**). Thus, the W144D mutein was selected as the lead candidate, as it not only mitigates oxidation liability but also presents the lowest predicted immunogenicity risk among the tested variants.

### 3.2 Assessment of W144D’s biological activity

Based on the *in silico* prediction results, we generated the W144D mutein to experimentally quantify its biological activity and immunogenicity risk. To assess whether the single point mutation W144D impacted the biological potency of the parent FLT3L-Fc protein, we used the human OCI-AML5 cell line, which has been reported to proliferate in response to human FLT3L stimulation (52, 53). Notably, both FLT3L-Fc-WT and W144D mutein induced similar dose-dependent proliferation of OCI-AML5 cells, with EC_50_ potency values of 6.0 pM and 6.3 pM, respectively (**Figure 2C**). This indicated that the W144D mutation did not significantly impact the molecule’s biological potency, thereby emerging as a promising candidate. To experimentally validate the low immunogenicity risk predicted for W144D, we used a suite of *in vitro* assays, including DC internalization, T cell proliferation, MAPPs and peptide binding assays.

### 3.3 DC internalization assessment

**Figure 3:**
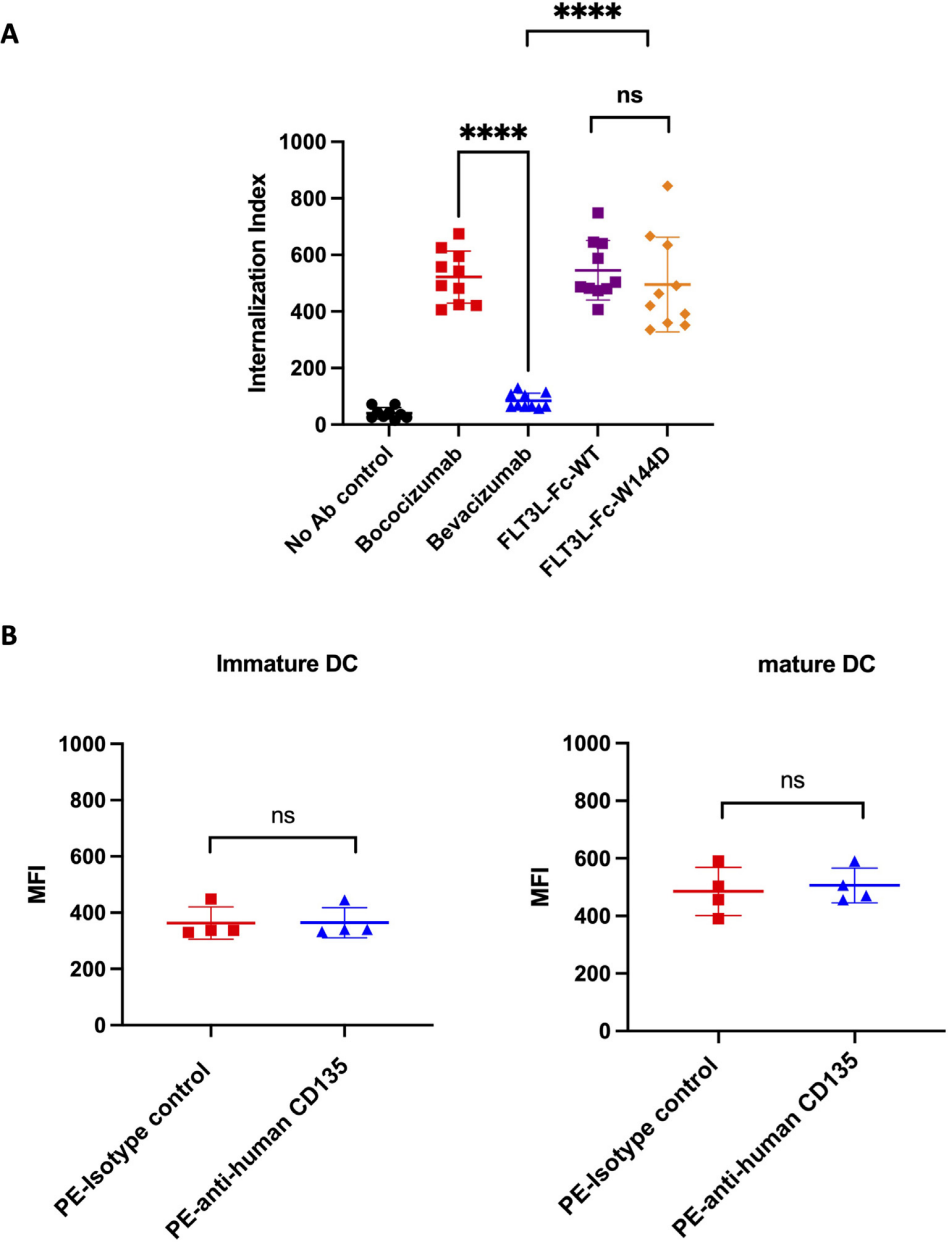
DC internalization assessment for FLT3L-Fc WT and W144D mutant. **(A)** Monocyte-derived dendritic cells from healthy donors (n = 10) were incubated with positive benchmark bococizumab with high ADA, negative benchmark bevacizumab with low ADA, FLT3L-Fc WT and W144D mutant. Internalization index is defined as the median fluorescence intensity (MFI) of internalized molecules detected by fluorescently labeled anti-human IgG secondary antibodies. **(B)** The expression of FLT3 receptor (CD135) on the surface of immature and mature monocyte-derived dendritic cells obtained from healthy donors (n = 4) was measured using PE labeled anti-human CD135 antibody. A PE labeled isotype control was used as a negative control. All data are mean ± SEM with pairwise comparisons (Wilcoxon matched-pairs signed rank test). ns, not significant, **** *P* ≤0.0001.

First, we assessed immunogenicity risk using a DC internalization assay. DC-based internalization assay has demonstrated a correlation between the magnitude of internalization and the immunogenicity risk of biotherapeutics (54). The characterization of internalization of FLT3L-Fc variants in monocyte derived DCs (MoDCs) was based on a previously reported assay (47), with the modification of using anti-human IgG (H+L) as the detection reagent. Two FLT3L-Fc variants were evaluated together with two control molecules: bococizumab (high internalization benchmark, clinical ADA 48%) and bevacizumab (low internalization benchmark, clinical ADA 0.6%) (55). As shown in **Figure 3A**, surprisingly, both FLT3L-Fc-WT and W144D showed very high levels of internalization, comparable to bococizumab. There was no statistical difference between WT and W144D mutein. Given that DCs can capture antigens through receptor-mediated endocytosis and/or non-specific constitutive macropinocytosis and considering that the FLT3 receptor is known to undergo rapid internalization upon dimerization by FLT3 ligand (56), it is important to examine the FLT3 receptor expression level on MoDCs to investigate the internalization mechanism. Interestingly, surface staining of both immature and mature MoDCs using flow cytometry did not reveal any detectable FLT3 receptor (**Figure 3B**), suggesting the high internalization of FLT3L-Fc variants in MoDCs is unlikely to be mediated by receptor-mediated endocytosis. Instead, the uptake may be facilitated by macropinocytosis, which plays a crucial role in DC mediated antigen presentation to CD4^+^ T cells. Nonetheless, the precise mechanism remains to be further elucidated.

### 3.4 T cell proliferation to assess immunogenicity risk

**Figure 4:**
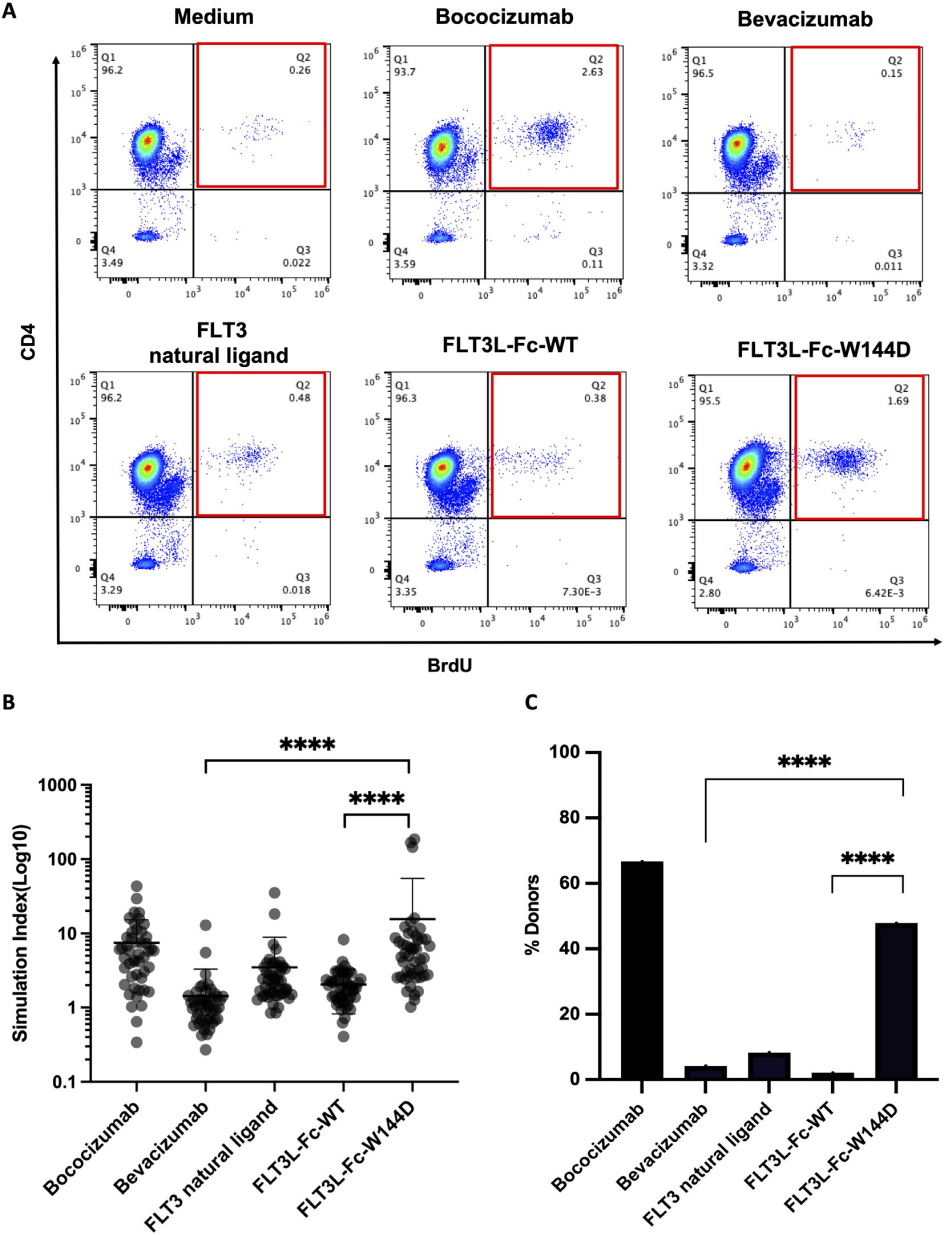
T cell proliferation to assess immunogenicity risk for FLT3L-Fc WT and W144D mutein. **(A)** Representative flow cytometry graphs of the percentage of CD4^+^BrdU^+^ T cells on CD3^+^ cells from a single donor for each condition. **(B)** Healthy donor-derived PBMCs (n = 48) were co-cultured with positive benchmark bococizumab with high ADA, negative benchmark bevacizumab with low ADA, FLT3 natural ligand, FLT3L-Fc-WT and FLT3L-Fc-W144D. Stimulation index (SI) is defined here as the ratio of the percentage of BrdU^+^CD4^+^CD3^+^ T cells from the treated group to BrdU^+^CD4^+^CD3^+^ T cells from the untreated group. **(C)** A positive response is defined as having an SI measurement that exceeds the cut-point value corresponding to the 95th percentile of the SI measurements for bevacizumab. The percentage of positive donors from different groups are shown. All data are mean ± SEM with pairwise comparisons (Wilcoxon matched-pairs signed rank test). **** P ≤0.0001.

Activation of T cells is a valuable marker for immunogenic responses, so we performed a reported PBMC-based CD4^+^ T cell proliferation assay (28) to further evaluate the immunogenicity risk for the two FLT3L-Fc variants. PBMCs isolated from 48 healthy donors, representing a diverse set of MHCII alleles (**Supplementary Table S2**) and exhibiting a broad genotype distribution of DRB1 alleles (**Supplementary Figure 1**), were stimulated with human natural FLT3 ligand, FLT3L-Fc-WT, and the W144D mutein. Additionally, bococizumab and bevacizumab served as positive and negative benchmarks, respectively. T cell proliferation was subsequently assessed by flow cytometry (**Figure 4A**). The stimulation index (SI) was calculated as the ratio of Brdu^+^CD4^+^ cells percent in the testing group over Brdu^+^CD4^+^ cells percent in the untreated group to define the positive response of the test molecules for each donor. As shown in **Figures 4B, C**, the positive control bococizumab induced a very high T cell response, with approximately 66% of donors testing positive, while the negative control bevacizumab exhibited a low stimulation index with around 5% positive donors. As expected, the natural ligand did not activate CD4^+^ T cells with only 8.3% of donors showing positive response. Interestingly, the results revealed a stark contrast between the two FLT3L-Fc variants. FLT3L-Fc-WT induced very low T cell response, comparable to bevacizumab and natural ligand, suggesting low immunogenic potential. Conversely, the W144D mutein elicited robust T cell responses, with around 48% of donors testing positive, indicating a high risk of immunogenicity. These findings suggest that while the W144D mutation does not impact the protein’s functional activity, it may introduce new epitopes capable of activating T cells, thereby increasing the risk of an immunogenic response. Furthermore, we analyzed the specific alleles from donors who exhibited positive responses to the W144D mutein. Interestingly, T cell activation induced by the W144D mutein was not limited to T cells associated with specific MHCII alleles. Instead, it occurred across T cells from donors with a diverse range of MHCII alleles as shown in **Supplementary Table S2**. These findings further underscore the increased risk of immunogenicity associated with the W144D mutein, but not the WT variant.

### 3.5 *In silico* prediction for new T cell epitopes

When the experimental assays were being performed, an updated pMHCII presentation prediction model was released, NetMHCIIpan-4.0. This model was shown to significantly outperform the initial model we used, NetMHCIIpan-3.2 (19, 35). Using NetMHCIIpan-4.0, both FLT3L-Fc-WT and W144D mutein were subsequently re-assessed for immunogenicity risk by searching the FLT3L region for potential immunogenic linear CD4^+^ T cell epitopes. The FLT3L sequence was computationally digested into all possible overlapping 12-20-mer peptides. A pMHCII pair was considered to be strongly presented if the elution percentile rank score was less than 10%. From these pMHCII pairs, the predicted binding cores were removed from further consideration if their sequences were identically aligned to 9-mer peptides from the human reference proteome (45). Among the remaining binding cores, those predicted to bind to more than one MHCII molecule were considered potentially immunogenic T cell epitopes. For FLT3L-Fc-WT, the FLT3L region did not have any potential immunogenic T cell epitopes predicted. In contrast, the same region in the W144D mutein was predicted to have two potential T cell epitopes, both of which overlapped with the mutated residue (**Table 1**), indicating a high immunogenicity risk for W144D. Compared to the previous prediction from NetMHCIIpan-3.2, the predictions from the newer pMHCII presentation model, NetMHCIIpan-4.0, were more consistent with the results from the T cell proliferation assay.

**Table 1:** *In silico*-predicted peptide cores in FLT3L-Fc-W144D.

FLT3L-Fc-W144D			
Predicted peptide core	Estimated global population coverage (%)	Allele	Predicted peptide
LVALKPDIT	55.95	HLA-DRB1*01:01	SEQLVALKPDITRQN
			TSEQLVALKPDITRQN
			SEQLVALKPDITRQ
			TSEQLVALKPDITRQ
			SEQLVALKPDITRQNF
		HLA-DRB1*04:01	SEQLVALKPDITRQN
			TSEQLVALKPDITRQN
			SEQLVALKPDITRQ
			TSEQLVALKPDITRQ
			SEQLVALKPDITRQNF
		HLA-DRB1*09:01	SEQLVALKPDITRQN
		HLA-DRB1*11:01	SEQLVALKPDITRQN
			TSEQLVALKPDITRQN
		HLA-DRB1*15:01	SEQLVALKPDITRQN
LKPDITRQN	30.99	HLA-DRB1*03:01	LVALKPDITRQNFSR
			QLVALKPDITRQNFSR
			EQLVALKPDITRQNFSR
			QLVALKPDITRQNFS
			VALKPDITRQNFSR
		HLA-DRB1*04:01	LVALKPDITRQNFSR
			QLVALKPDITRQNFSR
			VALKPDITRQNFSR
			LVALKPDITRQNFS
			EQLVALKPDITRQNFSR

Two potential immunogenic T cell epitopes in W144D. Each peptide-allele pair had an NetMHCIIpan-4.0 elution percentile rank score within the top 10%. Peptides are listed in order of strongest to weakest predicted binder (i.e., lowest to highest elution percentile rank score). For each binding core-allele pair with more than five peptides, only the top five peptides are shown.

### 3.6 MAPPs and *in vitro* peptide binding assay confirmed T cell epitopes presentation

**Figure 5:**
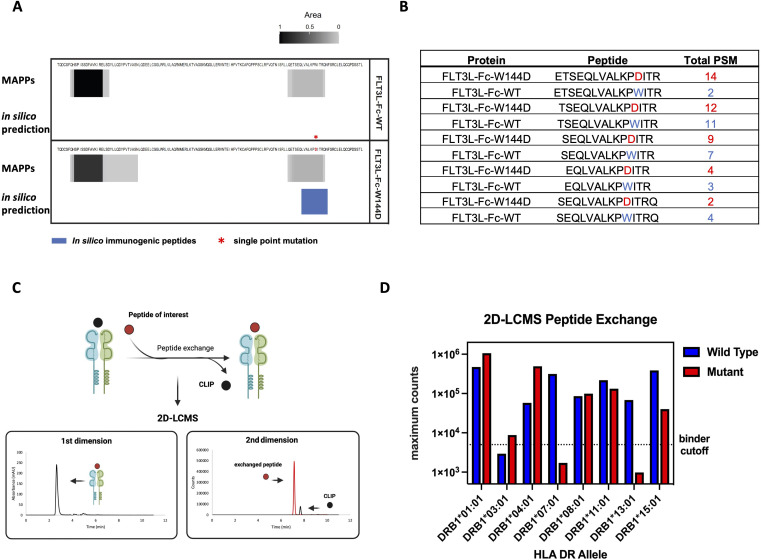
MAPPs and recombinant MHCII peptide binding assay to evaluate antigen specific peptides presentation **(A)** MAPPs peptide spectrum matches (PSMs) and *in silico* immunogenic predictions for FLT3L portion of two Fc fusion proteins. Peptide clusters detected across 10 human donors for each protein by MAPPs are collapsed into a single composite heatmap. The heatmap is scaled by area, which is normalized by dividing it by the maximum area from two Fc fusion proteins within the corresponding panel. Immunogenic T cell epitopes identified by NetMHCIIPan-4.0 are presented by solid blue bars. The immunogenic regions contain one or more 9-mer peptides with no matches in the human proteome and are predicted to bind two or more common HLA-DR alleles (percentile rank ≤ 10%). **(B)** Top 5 FLT3L-Fc-WT and W144D peptides detected by MAPPs spanning residue 144 are shown. The peptide ranking is based on the greatest number of PSMs observed in 10 donors, where >1 PSM is observed for at least 1 donor. Peptides selected for peptide exchange assay are based on these top 5 ranked FLT3L-Fc-W144D peptides. **(C)** Overview of 2D-LCMS workflow. In the first dimension, peptide-MHCII (pMHCII) complexes were separated from excess peptides in the reaction mixture using an analytical size exclusion column and then collected in a sampling loop. In the second dimension, the purified pMHCII complexes were injected into a reversed-phase column to separate the bound peptides from the MHCII complex to evaluate the binding efficiency between the peptides of interest and MHCII proteins. **(D)** 2D-LCMS extracted ion counts following peptide exchange across 8 common MHCII alleles. Wild-type (ETSEQLVALKPWITR) or W144D mutant (ETSEQLVALKPDITR) peptides reaching counts above the threshold (5e3) are considered true binders. The binding for both the wild type and mutant peptides indicates that these peptide sequences contain a promiscuous binding motif across many alleles.

To further evaluate the immunogenic potential of the FLT3L-Fc variants and specifically identify peptide regions from these variants bind to MHCII, we conducted a MAPPs assay. CD14^+^ monocytes were isolated from PBMCs of healthy human donors. Fifty percent of the donors (**Supplementary Table S3**) were selected based on sharing at least one HLA-DRB1 allele in common with donors that scored a positive T cell response in the T cell proliferation assay (**Figure 4**) and the other 50% of donors were selected randomly. The monocytes were subsequently differentiated into DCs. DCs were treated with FLT3L-Fc variants, matured, and lysed. HLA-DR molecules were immunoprecipitated from the lysates and peptides bound to HLA-DR proteins were acid eluted from the pMHCII complex and detected by mass spectrometry. MAPPs enabled the identification and relative quantification of HLA-DR presented peptides corresponding to the FLT3L-Fc variants, thus gaining a deeper understanding of their immunogenic potential. Similar peptide clusters were detected for both FLT3L-Fc-WT, natural ligand, and W144D mutein (**Supplementary Figure 2A**). Peptide clusters containing the W144 residue in both the WT and W144D mutein were detected by MAPPs (**Figure 5A**). Interestingly, two peptide cores (LVALKPDIT and LKPDITRQN) identified by *in silico* prediction (**Table 1**) overlapped with the peptides detected by MAPPs in the W144D mutein. Comparisons between WT and mutein proteins showed broadly overlapping peptide clusters in the mutation region, but with more peptide spectrum matches (PSM) in the FLT3L-Fc-W144D variant (**Figure 5B**). Furthermore, presentation of peptide clusters containing W144D was only observed in donors 1, 6, 7 and 10. Donors 6, 7, and 10 share the HLA-DRB1*01:01 allele (**Supplementary Figure 2B**), which was also found in some donors who had positive T cell responses in the T cell proliferation assay. Although we observed a 3-5x greater mass spectrometry signal for the peptide ETSEQLVALKPDIRT (W144D) compared to ETSEQLVALKPWIRT (WT) in donors 6 and 10, as determined by area-under-the-curve (AUC) of the corresponding peak (**Supplementary Figure 2C**), it was difficult to establish a statistically significant correlation between MHCII alleles with positive T cell responses and MAPPs from our correlation analysis.

Next, we performed two-dimensional liquid chromatography-mass spectrometry (2D-LCMS) to further evaluate the efficiency of *in vitro* peptide exchange with MHCII proteins and peptides of interest. To evaluate peptide binding across multiple common MHCII alleles, we used recombinant MHCII proteins containing an exchangeable CLIP peptide variant. These proteins were exposed to low pH conditions to enable the exchange of the CLIP peptide with the epitope peptides. Peptide exchange was monitored using 2D-LCMS, and the propensity of each peptide to bind the MHCII allele of interest was determined by comparison of the mass spec peak intensities for the exchanged peptides. In the first dimension, pMHCII complexes were separated on an analytical size exclusion column from excess peptides in the reaction mixture and then collected in a sampling loop. In the second dimension, the purified pMHCII collected in the sampling loop was injected onto a reversed-phase column to separate bound peptides from the MHCII complex. These steps enabled us to evaluate the binding efficiency between the MHCII proteins and the peptides of interest (**Figure 5C**). The top pair of peptides, ETSEQLVALKPWITR and ETSEQLVALKPDITR, identified from the MAPPs assay for the WT and W144D mutein, respectively, were synthesized as representatives and examined using 2D-LCMS analysis. The results demonstrated that both the WT peptide and the peptide containing the W144D mutation promiscuously bind to MHCII alleles (**Figure 5D**; **Supplementary Figure 3**).

Of the eight MHCII alleles tested, the highest recovery of complex and mass spec intensity for W144D was observed in DRB1*01:01 and DRB1*04:01; for which only DRB1*01:01 was the same donor MHCII allele that W144D T cell epitopes were detected by MAPPs. Taken together, these findings suggest stronger peptide binding is not always directly proportional to greater presentation for all epitopes. Additionally, the peptide binding data and T cell assay results indicate that T cells specific to the WT sequence are likely tolerized or clonally deleted during development. In contrast, the T cell response to the W144D mutein suggests that the D mutation is recognized as a foreign antigen by cognate TCRs, thereby inducing a strong T cell response and enhancing immunogenicity risk.

### 3.7 *In silico* predicted T cell epitopes are homologous to microbial sequences

In addition to the increased immunogenicity risk associated with a higher number of T cell epitopes, some T cell epitopes can carry additional risk due to their similarity to epitopes present on microbial agents (57). To understand the potential mechanism by which a single point mutation could introduce new T cell epitopes that significantly enhance the immunogenicity response, we conducted a sequence similarity alignment against bacteria proteins using NCBI protein BLAST (Basic Local Alignment Search Tool). One of the *in silico* predicted epitopes, LVALKPDIT, was identical to sequences from 2 bacterial strains. In addition, this epitope included 8-mer subsequences (VALKPDIT and LVALKPDI) that were identical to sequences from 70 different bacterial strains (**Supplementary Figure 4**; **Supplementary Table S4**). Among these, approximately 41% of the strains belonged to the Pseudomonadota family, a major phylum of Gram-negative bacteria found in human gut microbiota (58). In contrast, no bacterial sequences were identical to the corresponding sequence (LVALKPWIT) in the WT molecule. This finding may explain the high immunogenic response induced by the W144D mutein.

## 4 Discussion

In this study, we employed a suite of assessment tools to evaluate the immunogenicity risk for FLT3L-Fc-WT and the W144D mutein during the lead selection and optimization stage. Both FLT3L-Fc-WT and W144D exhibited very high levels of internalization by DCs, while the FLT3L-W144D mutein, but not the WT variant, induced strong promiscuous T cell responses, indicating a high immunogenicity potential. An updated *in silico* prediction method identified a potential conserved motif of peptide presentation around W144. No potential immunogenic T cell epitopes were identified in the sequence of FLT3L-Fc-WT. Peptides containing the W144D site were further confirmed to bind to common HLA-DRB1 alleles by MAPPs and recombinant MHCII-peptide binding assay. Additionally, the identified peptide showed an identical match to sequence of proteins from different bacterial strains, which might be the root cause for the high immunogenicity risk induced by a single point mutation. Consequently, the mutein variant was removed from further consideration as a clinical candidate. These studies revealed that a single point mutation on a human self-ligand could introduce highly immunogenic T-cell epitopes. The combination of these assessment tools provided us with a comprehensive understanding of the immunogenicity risk associated with the W144D FLT3L-Fc mutein, which guided our decision-making process. This clearly demonstrates the importance of a comprehensive and integrated strategic approach for the immunogenicity risk assessment in the development of biotherapeutics.

As the production of ADAs may affect both the efficacy and safety of the drug, immunogenicity becomes a critical concern in the development of biotherapeutics, including antibodies and Fc fusion proteins (59). While Fc-fusion proteins are designed to extend their half-life and improve PK *in vivo*, a significant body of immunological literature has shown that the strategic design of the Fc component can engage and modulate the immune response, including immunogenicity (60–62). One consideration is that the joining region in the Fc fusion protein may introduce undesired formation of neoantigens, thereby increasing the immunogenicity risk (60). Additionally, the interaction between Fc domain and its receptors further exacerbates this concern, as crosslinking of activating Fc gamma receptors (FcγR) on APCs facilitates antigen uptake and maturation, which is one of the critical steps for the ADA production (63). To mitigate this risk and for other purposes, we generated a FLT3L-Fc fusion protein with attenuated Fc effector functions to improve its safety profile (40). While we observed high DC internalization for both WT and W144D mutein, only the mutein induced significant T cell proliferation, whereas the WT variant did not. We investigated the potential mechanism underlying the high DC internalization levels of both variants by examining the surface expression level of the FLT3 receptor. No receptor was detected on either immature or mature DCs, indicating that the internalization was not receptor-mediated endocytosis. The low T cell response induced by the WT variant also suggests that the joining region between the ligand and Fc did not introduce neoantigens. This was further confirmed by *in silico* predictions, which showed that new T cell epitopes were only identified in the mutation regions containing W144D.

In this study, we identified potential CD4^+^ T cell epitopes in the FLT3L region using two pMHCII prediction models. Compared to NetMHCIIpan-3.2, we found that NetMHCIIpan-4.0 predictions better aligned with the results from the experimental assays, which showed a high immunogenicity risk for the W144D mutein. It suggests that the predictive ability of *in silico* models like NetMHCIIpan can be significantly improved by training on sufficient experimental data when available, leading to more accurate identification of potential immunogenic epitopes and better risk assessment in the development of biotherapeutics.

Notably, the potential epitopes predicted *in silico* overlapped with the peptide clusters observed in MAPPs experiments. This concordance between experimental assays and computational predictions increases our confidence that this region indeed contains the epitope responsible for the immunogenic response observed in the T cell proliferation assay. When *in silico* prediction is integrated with MAPPs, there is a positive correlation between the number of potential T cell epitopes and immunogenicity risk (28). This correlation suggests that computational models like NetMHCIIpan-4.0 can reliably predict regions of high immunogenic potential, which can then be experimentally validated through MAPPs and T cell proliferation assay. The ability to cross-validate predictions with experimental data not only strengthens the predictive power of the computational models but also provides a comprehensive framework for assessing immunogenicity risk. By combining the high-throughput capabilities of *in silico* predictions with the specificity of experimental assays, we can more accurately identify and characterize epitopes that may contribute to adverse immune responses in the early development stages of drug candidates. This dual validation process is crucial for the rational design and optimization of biotherapeutics, ensuring both efficacy and safety.

A number of studies have shown that point mutations on the T cell epitopes have great potential to alter CD4^+^/CD8^+^ T cell responses (64). While cancer cells or pathogens have the inherent ability to escape recognition by TCRs through mutation of immunogenic epitopes (65), highly engineered protein therapeutics undergo multiple mutation processes to achieve better molecular properties. However, these mutations could also increase the potential to introduce new T cell epitopes recognized by immune cells, thereby enhancing immunogenicity risk of the therapeutics (66). As characterized in this study, a single point mutation on the FLT3 ligand protein introduced new T cell epitopes and induced a promiscuous T cell response with high immunogenicity potential. A recent study showed a seven–amino acid sequence (FIDPDDD) from brolucizumab, an anti-VEGF biotherapeutic, was identical to a sequence found in human gut bacterial proteins. This biotherapeutic has been associated with ADA responses in 40 to 75% of patients and healthy individuals. The study suggested that exposure of the immune system to such bacteria could potentially trigger an immune response and antibody production against the bacterial proteins that overlap with the FIDPDDD sequence from brolucizumab (57), thereby enhancing ADA response. Interestingly, we found similar results in our study. The identified T cell epitope core sequence LVALKPDIT from the W144D mutein was conserved across different bacterial proteins from various strains, including a bacterial family found in human gut microbiota, while the corresponding WT sequence LVALKPWIT did not. This suggests a potential root cause for why the single point mutation could enhance the immunogenicity response. Exposure to these bacterial proteins could prime the immune system to recognize and respond to the mutated epitope, thereby increasing the risk of immunogenicity. These findings suggest conducting homology sequence searches for the identified epitopes could be highly valuable for immunogenicity risk assessment during the lead identification stage. This approach can provide critical insights into the potential cross-reactivity with microbial antigens, thereby informing the design and optimization of biotherapeutics to minimize immunogenicity risk.

In this study, we observed that peptide clusters containing the W144D mutation site were presented in donors with specific alleles, such as HLA-DRB1*01:01, as identified by the MAPPs assay. Furthermore, the highest recovery of peptide-MHCII complexes and mass spectrometry intensity for the W144D variant was observed in DRB1*01:01 and DRB1*04:01 among the eight MHCII alleles tested in the peptide binding assay. Interestingly, T cell activation induced by the W144D mutein was not restricted to donors with one or two specific MHCII alleles but was observed across donors with a diverse range of MHCII alleles, including DRB1*01:01 and DRB1*04:01. However, subsequent correlation analysis indicated that establishing a statistically significant correlation between MHCII alleles and positive T cell responses, as well as peptide presentation, was challenging. This limitation may be due to the restricted number of donors included in these *in vitro* assays.

In summary, this study demonstrates that even a single point mutation can introduce new T cell epitopes, significantly altering the immunogenic profile of a protein. This finding emphasizes the need for careful consideration of mutations during the engineering of biotherapeutics. It underscores the value of using advanced *in silico* prediction models, such as NetMHCIIpan-4.0, to identify potential immunogenic epitopes in combination with experimental assays applied in this case study. The positive correlation between computational predictions and experimental data highlights the importance of using a multifaceted approach to immunogenicity assessment in the development of biotherapeutics.

## Data Availability

The MAPPs data presented in the study are deposited in the MASSive repository, accession number MSV000096859.
