# Supplementary material for: A single point mutation on FLT3L-Fc protein increases the risk of immunogenicity
Source: Front Immunol. 2025 Feb 13;16:1519452. doi: 10.3389/fimmu.2025.1519452 (PMC11865242; doi:10.3389/fimmu.2025.1519452)
Supplement: Supplementary file 1 [file Table1.docx]

**Supplementary Tables**

**A single point mutation on FLT3L-Fc protein increases the risk of Immunogenicity**

Dan Qin^1#^, Qui Phung^2#^, Patrick Wu^3#^, Zhaojun Yin^3^, Sien Tam^1^, Peter Tran^3^, Adel Elsohly^4^, Joshua Gober^4^, Zicheng Hu^3^, Zhenru Zhou^2^, Sivan Cohen^3^, Dongping He^1^, Travis W. Bainbridge^4^, Christopher C. Kemball^1^, Jonathan Zarzar^5^, Alavattam Sreedhara^5^, Nicole Stephens^6^, Jérémie Decalf^7^, Christine Moussion^7^, Zhengmao Ye^1^, Mercedesz Balazs^1^, Yinyin Li^1*^

^1^Biochemical and Cellular Pharmacology, ^2^Proteomic and Genomic Technologies, ^3^BioAnalytical Sciences, ^4^Protein Chemistry, ^5^Pharmaceutical Development, ^6^Analytical Development & Quality Control, ^7^Cancer Immunology

Genentech Inc., South San Francisco, United States.

^#^These authors have contributed equally to this work

*CONTACT: liy416@gene.com

Department of Biochemical and Cellular Pharmacology, Genentech, Inc. South San Francisco, CA, USA

**KEYWORDS**

Anti-drug antibodies; immunogenicity; FLT3L-Fc; *in silico* prediction; *In vitro* cellular assay; T cell proliferation; DC internalization; MAPPs; T cell epitope.

**Table S1**. Amino acid sequences of FLT3L-Fc-WT and FLT3L-Fc-W144D mutein

| **Name** | **Sequence** |
| --- | --- |
| FLT3L-Fc-WT | TQDCSFQHSPISSDFAVKIRELSDYLLQDYPVTVASNLQDEELCGGLWRLVLAQRWMERLKTVAGSKMQGLLERVNTEIHFVTKCAFQPPPSCLRFVQTNISRLLQETSEQLVALKPWITRQNFSRCLELQCQPDSSTLDKTHTCPPCPAPPVAGPSVFLFPPKPKDTLMISRTPEVTCVVVDVSHEDPEVKFNWYVDGVEVHNAKTKPREEQYGSTYRVVSVLTVLHQDWLNGKEYKCKVSNKALPAPIEKTISKAKGQPREPQVYTLPPSREEMTKNQVSLTCLVKGFYPSDIAVEWESNGQPENNYKTTPPVLDSDGSFFLYSKLTVDKSRWQQGNVFSCSVMHEALHNHYTQKSLSLSPGK |
| FLT3L-Fc-W144D | TQDCSFQHSPISSDFAVKIRELSDYLLQDYPVTVASNLQDEELCGGLWRLVLAQRWMERLKTVAGSKMQGLLERVNTEIHFVTKCAFQPPPSCLRFVQTNISRLLQETSEQLVALKPDITRQNFSRCLELQCQPDSSTLDKTHTCPPCPAPPVAGPSVFLFPPKPKDTLMISRTPEVTCVVVDVSHEDPEVKFNWYVDGVEVHNAKTKPREEQYGSTYRVVSVLTVLHQDWLNGKEYKCKVSNKALPAPIEKTISKAKGQPREPQVYTLPPSREEMTKNQVSLTCLVKGFYPSDIAVEWESNGQPENNYKTTPPVLDSDGSFFLYSKLTVDKSRWQQGNVFSCSVMHEALHNHYTQKSLSLSPGK |

**Table S2.** HLA genotypes for donors used in T cell proliferation assay and T cell response outcome for each donor. NA, allele not found or insufficient data to determine the allele.

| Donor | Allele_1_DRB1 | Allele_1_DPB1 | Allele_1_DQB1 | Allele_2_DRB1 | Allele_2_DPB1 | Allele_2_DQB1 | T_Cell_response |
| --- | --- | --- | --- | --- | --- | --- | --- |
| 84 | DRB1*09:01:02:01 | DPB1*04:02:01:02 | DQB1*03:03:02:02 | DRB1*09:01:02:01 | DPB1*05:01:01:01 | DQB1*03:03:02:02 | Positive |
| 85 | DRB1*07:01:01:01 | DPB1*02:01:02:01 | DQB1*02:02:01:01 | DRB1*13:02:01:02 | DPB1*05:01:01:01 | DQB1*06:04:01:01 | Negative |
| 86 | DRB1*11:01:01:01 | DPB1*02:01:02:01 | DQB1*03:01:01:02 | DRB1*14:54:01:01 | DPB1*04:02:01:01 | DQB1*03:01:01:19 | Positive |
| 87 | DRB1*04:01:01:01 | DPB1*04:01:01:01 | DQB1*02:02:01:01 | DRB1*07:01:01:01 | DPB1*04:01:01:01 | DQB1*03:01:01:01 | Positive |
| 88 | DRB1*08:03:02:01 | DPB1*02:01:02:01 | DQB1*03:01:01:02 | DRB1*13:05:01 | DPB1*04:01:01:01 | DQB1*06:01:01:01 | Negative |
| 89 | DRB1*01:02:01:01 | DPB1*04:01:01:10 | DQB1*03:01:01:02 | DRB1*11:01:01:01 | DPB1*04:01:01:27 | DQB1*05:01:01:01 | Positive |
| 90 | DRB1*01:01:01:01 | DPB1*04:01:01:01 | DQB1*02:01:01 | DRB1*03:01:01:01 | DPB1*04:02:01:02 | DQB1*05:01:01:02 | Negative |
| 91 | DRB1*04:03:01:01 | DPB1*04:01:01:01 | DQB1*03:01:01:02 | DRB1*13:05:01 | DPB1*04:01:01:01 | DQB1*03:05:01 | Positive |
| 92 | DRB1*09:01:02:01 | DPB1*02:02:01:01 | DQB1*03:03:02:02 | DRB1*14:05:01:01 | DPB1*135:01 | DQB1*05:03:01:01 | Negative |
| 93 | NA | NA | NA | NA | NA | NA | Positive |
| 94 | DRB1*04:04:01 | DPB1*04:01:01:01 | DQB1*03:05:01 | DRB1*13:02:01:02 | DPB1*04:01:01:01 | DQB1*06:09:01:01 | Negative |
| 95 | DRB1*15:01:01:01 | DPB1*02:02:01:01 | DQB1*05:02:01:01 | DRB1*15:01:01:01 | DPB1*13:01:01:02 | DQB1*06:01:01:01 | Negative |
| 96 | DRB1*04:01:01:01 | DPB1*03:01:01:01 | DQB1*03:01:01:04 | DRB1*11:01:01:01 | DPB1*04:01:01:01 | DQB1*03:02:01:01 | Negative |
| 97 | DRB1*03:01:01:01 | DPB1*17:01:01:01 | DQB1*02:01:01 | DRB1*12:02:01:01 | DPB1*19:01:01:01 | DQB1*03:01:01:12 | Negative |
| 98 | DRB1*11:03:01 | DPB1*02:01:02:01 | DQB1*03:01:01:02 | DRB1*15:01:01:01 | DPB1*04:01:01:03 | DQB1*06:02:01:01 | Negative |
| 99 | DRB1*08:03:02:01 | DPB1*02:02:01:01 | DQB1*03:01:01:02 | DRB1*11:01:01:01 | DPB1*05:01:01:01 | DQB1*06:01:01:01 | Positive |
| 100 | DRB1*12:02:01:01 | DPB1*05:01:01:01 | DQB1*03:01:01:07 | DRB1*15:01:01:01 | DPB1*13:01:01:02 | DQB1*06:01:01:01 | Negative |
| 101 | DRB1*07:01:01:01 | DPB1*02:01:02:10 | DQB1*03:03:02:01 | DRB1*15:01:01:01 | DPB1*02:01:02:10 | DQB1*06:02:01:01 | Negative |
| 102 | DRB1*07:01:01:01 | DPB1*417:01:01 | DQB1*02:02:01:01 | DRB1*15:01:01:01 | DPB1*849:01 | DQB1*06:02:01:01 | Positive |
| 103 | DRB1*03:01:01:01 | DPB1*04:01:01:01 | DQB1*02:01:01 | DRB1*15:01:01:01 | DPB1*04:02:01:02 | DQB1*06:02:01:01 | Positive |
| 104 | NA | NA | NA | NA | NA | NA | Negative |
| 105 | DRB1*12:02:01:01 | DPB1*02:01:02:01 | DQB1*03:01:01:12 | DRB1*15:02:01:01 | DPB1*02:01:02:11 | DQB1*05:01:24:01 | Negative |
| 106 | NA | NA | NA | NA | NA | NA | Positive |
| 107 | DRB1*07:01:01:01 | DPB1*02:02:01:01 | DQB1*02:02:01:01 | DRB1*11:01:01:01 | DPB1*17:01:01:01 | DQB1*03:01:01:02 | Negative |
| 108 | DRB1*13:02:01:02 | DPB1*04:01:01:01 | DQB1*03:01:01:02 | DRB1*13:03:01 | DPB1*10:01:01:01 | DQB1*06:04:01:01 | Negative |
| 109 | DRB1*03:01:01:01 | DPB1*02:02:01:01 | DQB1*02:01:01 | DRB1*15:01:01:01 | DPB1*04:01:01:01 | DQB1*06:02:01:01 | Negative |
| 110 | DRB1*04:02:01 | DPB1*04:01:01:03 | DQB1*03:02:01:01 | DRB1*13:01:01:01 | DPB1*10:01:01:01 | DQB1*06:03:01:01 | Positive |
| 111 | DRB1*04:01:01:01 | DPB1*03:01:01:01 | DQB1*03:02:01:01 | DRB1*08:01:01 | DPB1*04:01:01:01 | DQB1*04:02:01:01 | Positive |
| 112 | DRB1*03:01:01:01 | DPB1*02:01:02:10 | DQB1*02:01:01 | DRB1*07:01:01:01 | DPB1*15:01:01:01 | DQB1*02:02:01:01 | Positive |
| 113 | DRB1*08:01:01 | DPB1*04:01:01:01 | DQB1*03:01:01:01 | DRB1*14:02:01:01 | DPB1*04:02:01:02 | DQB1*04:02:01:01 | Positive |
| 114 | DRB1*04:07:01:01 | DPB1*04:02:01:02 | DQB1*03:02:01:01 | DRB1*04:11:01 | DPB1*04:02:01:02 | DQB1*04:02:01:09 | Negative |
| 115 | DRB1*07:01:01:01 | DPB1*02:01:02:10 | DQB1*03:03:02:01 | DRB1*14:54:01:01 | DPB1*05:01:01:03 | DQB1*05:03:01:01 | Negative |
| 116 | NA | NA | NA | NA | NA | NA | Positive |
| 117 | NA | NA | NA | NA | NA | NA | Negative |
| 118 | NA | NA | NA | NA | NA | NA | Negative |
| 119 | DRB1*03:01:01:01 | DPB1*04:01:01:01 | DQB1*02:01:01 | DRB1*04:01:01:01 | DPB1*04:01:01:01 | DQB1*03:01:01:01 | Positive |
| 120 | DRB1*01:01:01:01 | DPB1*04:01:01:10 | DQB1*03:01:01:05 | DRB1*12:01:01:01 | DPB1*04:02:01:02 | DQB1*05:01:01:02 | Negative |
| 121 | DRB1*03:01:01:01 | DPB1*09:01:01 | DQB1*02:01:01 | DRB1*04:05:01:01 | DPB1*104:01:01:01 | DQB1*03:02:01:01 | Negative |
| 122 | DRB1*11:01:01:01 | DPB1*04:01:01:01 | DQB1*03:01:01:02 | DRB1*15:01:01:01 | DPB1*05:01:01:01 | DQB1*06:02:01:01 | Positive |
| 123 | DRB1*12:02:01:01 | DPB1*05:01:01:01 | DQB1*03:01:01:12 | DRB1*13:02:01:01 | DPB1*09:01:01 | DQB1*06:09:01:01 | Negative |
| 125 | DRB1*12:02:01:01 | DPB1*05:01:01:01 | DQB1*03:01:01:07 | DRB1*15:01:01:01 | DPB1*21:01 | DQB1*06:02:01:01 | Negative |
| 126 | DRB1*11:01:01:01 | DPB1*02:01:02:01 | DQB1*03:01:01:02 | DRB1*15:01:01:01 | DPB1*04:01:01:03 | DQB1*06:02:01:01 | Positive |
| 127 | DRB1*01:01:01:01 | DPB1*01:01:01:01 | DQB1*02:02:01:02 | DRB1*09:01:02:01 | DPB1*17:01:01:01 | DQB1*05:01:01:02 | Positive |
| 129 | DRB1*03:01:01:01 | DPB1*04:01:01:01 | DQB1*02:01:01 | DRB1*15:01:39 | DPB1*04:01:01:03 | DQB1*06:02:01:01 | Negative |
| 130 | DRB1*04:02:01 | DPB1*04:01:01:01 | DQB1*03:02:01:01 | DRB1*04:02:01 | DPB1*04:01:01:01 | DQB1*03:02:01:01 | Negative |
| 132 | DRB1*03:01:01:01 | DPB1*04:01:01:01 | DQB1*02:01:01 | DRB1*14:54:01:01 | DPB1*26:01:02 | DQB1*05:03:01:01 | Positive |
| 133 | DRB1*04:01:01:01 | DPB1*04:01:01:01 | DQB1*03:02:01:01 | DRB1*15:02:01:01 | DPB1*04:02:01:01 | DQB1*06:01:01:01 | Positive |
| 134 | DRB1*01:01:01:01 | DPB1*416:01:01:01 | DQB1*02:01:01 | DRB1*03:01:01:01 | DPB1*665:01 | DQB1*05:01:01:02 | Positive |

**Table S3.** HLA genotypes for donors used in MAPPs assay.

| Donor | Allele_1_DRB1** | Allele_1_DPB1 | Allele_1_DQB1 | Allele_2_DRB1 | Allele_2_DPB1 | Allele_2_DQB1 |
| --- | --- | --- | --- | --- | --- | --- |
| 1 | 03:01:01G | 04:01:01G | 02:01:01G | 07:01:01G | 04:02:01G | 02:02:01 |
| 2 | 07:01:01G | 04:01:01G | 02:02:01 | 13:02:01 | 04:02:01G | 06:04:01G |
| 3 | 13:01:01G | 04:01:01G | 06:01:01G | 15:02:01G | 20:01:01 | 06:03:01G |
| 4 | 11:01:01G | 04:01:01G | 03:01:01G | 11:04:01G | 04:02:01G | 03:01:01G |
| 5 | 04:01:01G | 02:01:02G | 03:01:01G | 11:04:01 | 03:01:01G | 03:01:01G |
| 6 | 01:01:01 | 03:01:01G | 05:01:01G | 13:01:01G | 04:01:01G | 06:03:01G |
| 7 | 01:01:01 | 01:01:01G | 05:01:01G | 15:02:01G | 13:01:01G | 06:01:01G |
| 8 | 03:01:01G | 01:01:01G | 02:01:01G | 11:01:01G | 04:01:01G | 03:01:01G |
| 9 | 11:01:01G | 02:01:02G | 03:01:01G | 13:02:01G | 05:01:01G | 06:04:01G |
| 10 | 01:01:01G | 03:01:01G | 02:01:01G | 03:01:01G | 04:01:01G | 05:01:01G |

** From 01 April 2010, all groups of HLA alleles that have identical nucleotide sequences across the exons encoding the peptide binding domains (exon 2 for HLA class II alleles) are designated by an uppercase ‘G’ which follows the three-field allele designation of the lowest numbered allele in the group. A full list of these groups can be seen at http://hla.alleles.org/nomenclature/g_groups.html.

**Table S4.** Species associated with the top 70 hits identified in a homology search with the FLT3L-Fc-W144D peptide sequence LVALKPDIT.

| **Taxonomic assignment of top 70 hits for LVALKPDIT sequence** | |
| --- | --- |
| Acidimicrobiales bacterium | Nitrosospira briensis |
| Alphaproteobacteria bacterium | Nitrospirales bacterium |
| Bacillota bacterium | Pasteurella multocida subsp. multocida |
| Bacteroidetes bacterium ADurb.BinA174 | Planctomycetes bacterium RIFCSPHIGHO2_12_FULL_52_36 |
| Bradyrhizobium ottawaense | Planctomycetota bacterium |
| Bradyrhizobium sp. | Polyangiaceae bacterium |
| Burkholderiaceae bacterium | Proteiniphilum sp. |
| candidate division NPL-UPA2 bacterium | Sphingobacteriales bacterium |
| Chitinophagaceae bacterium | Sphingobacteriales bacterium 44-61 |
| Eubacterium sp. | Streptomyces sp. TLI_55 |
| Filimonas zeae | Syntrophobacterales bacterium |
| Geobacteraceae bacterium | Thermodesulforhabdaceae bacterium |
| Holophaga sp. | uncultured Draconibacterium sp. |
| Longimicrobiales bacterium | uncultured Ruegeria sp. |
| Lysobacteraceae bacterium | Verrucomicrobiota bacterium |
| Mesorhizobium australicum | Vibrio japonicus |
| Mesorhizobium sp. M0051 | Vibrio sp. CAU 1672 |
| Methylococcales bacterium | Vibrio sp. PID17_43 |
| Mycobacterium sp. | Vibrio sp. PID23_8 |
