# Supplementary figures and images for: A single point mutation on FLT3L-Fc protein increases the risk of immunogenicity
Source: Front Immunol. 2025 Feb 13;16:1519452. doi: 10.3389/fimmu.2025.1519452 (PMC11865242; doi:10.3389/fimmu.2025.1519452)

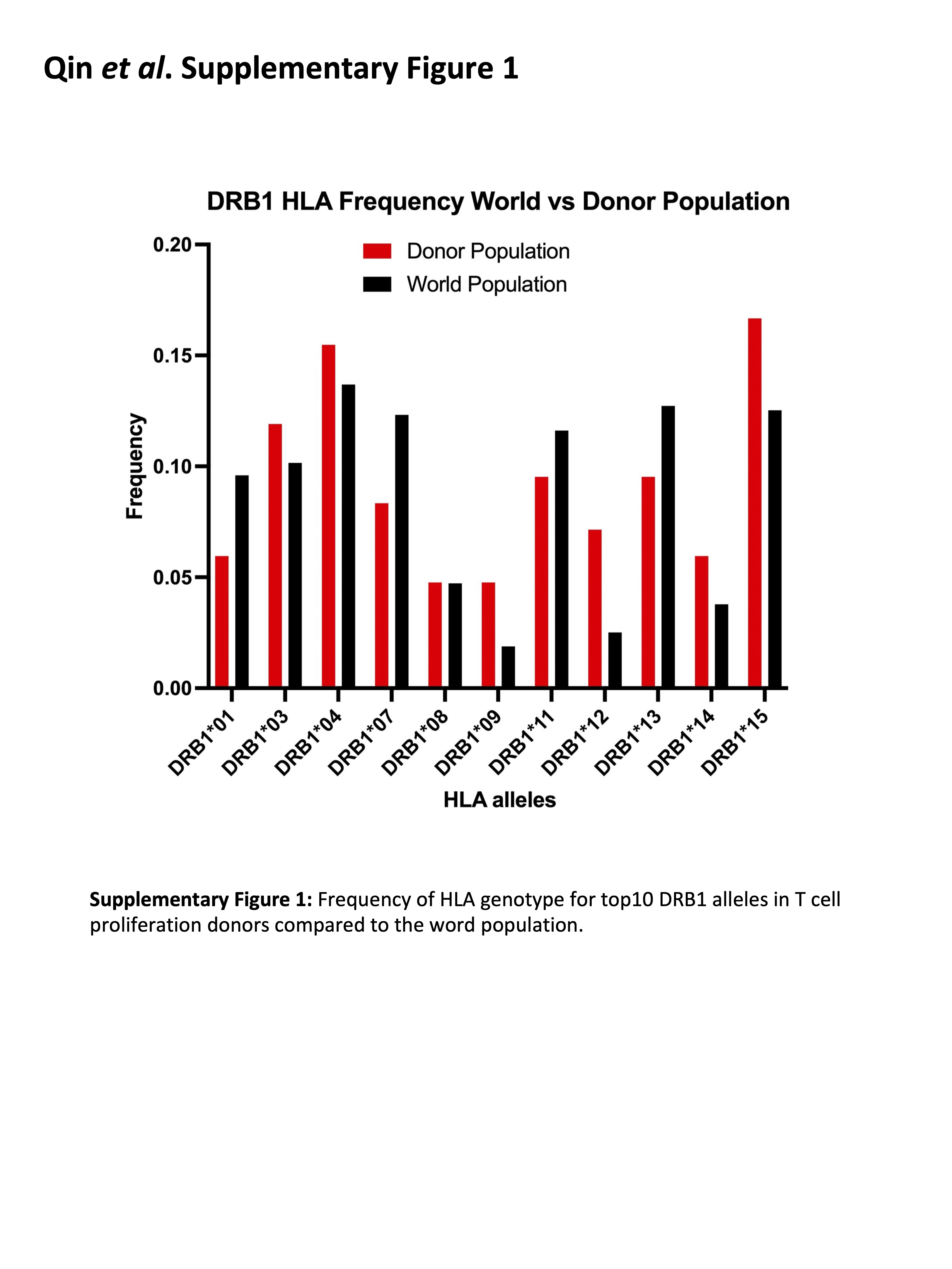

Supplement: Supplementary file 2 [file Image1.jpg]

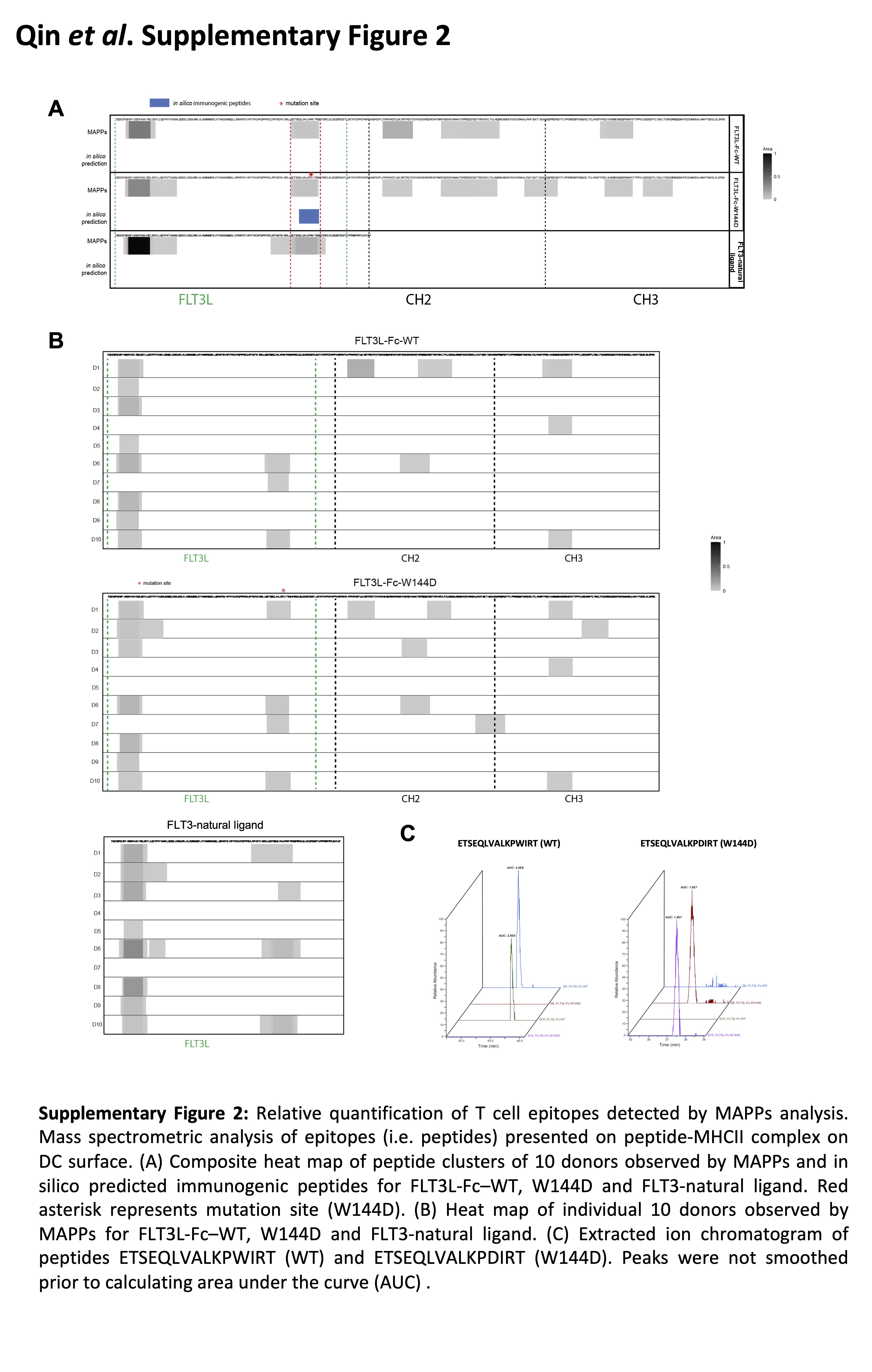

Supplement: Supplementary file 3 [file Image2.jpg]

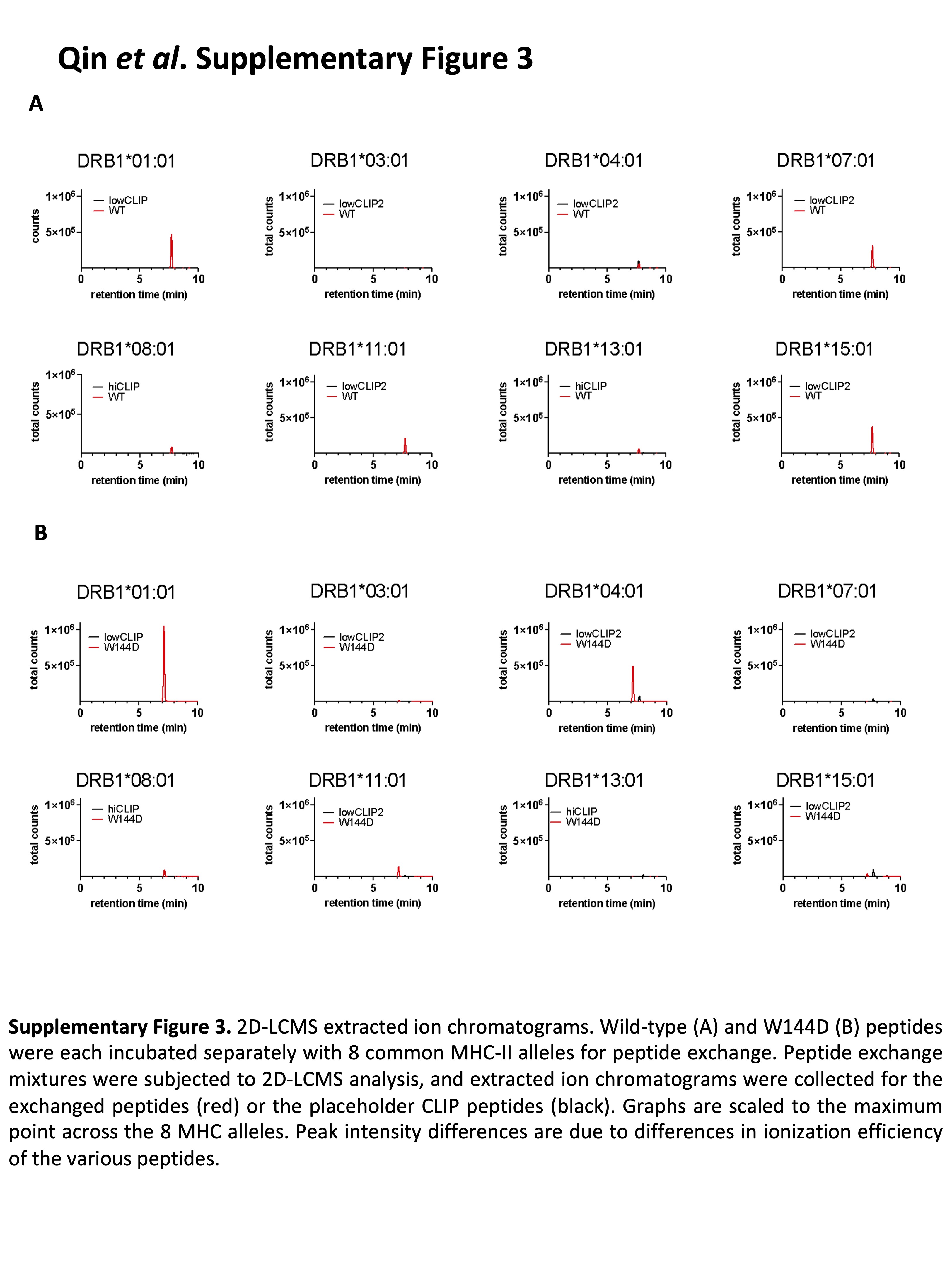

Supplement: Supplementary file 4 [file Image3.jpg]

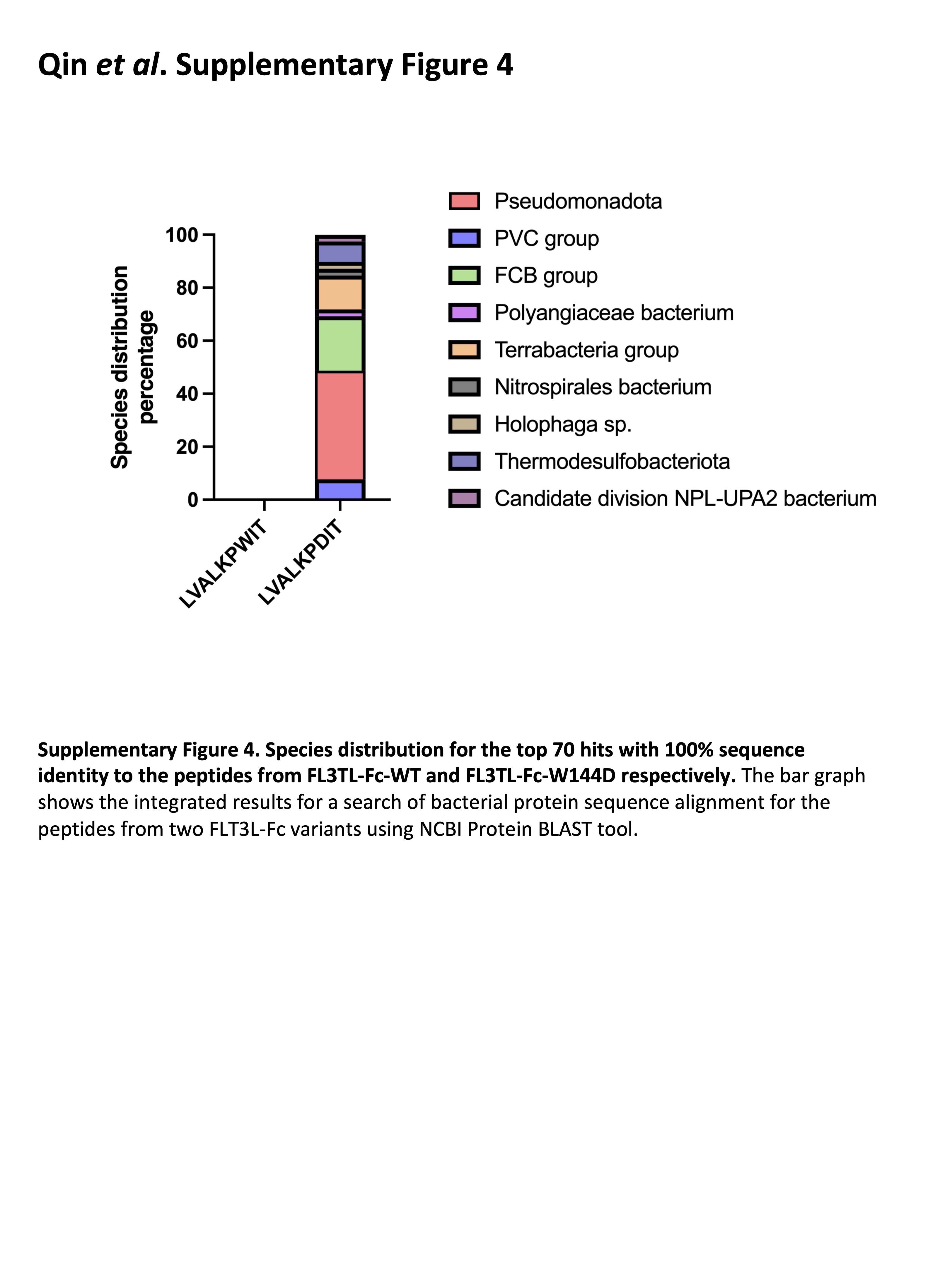

Supplement: Supplementary file 5 [file Image4.jpg]
